# Design, Manufacture and Testing of Capacitive Pressure Sensors for Low-Pressure Measurement Ranges

**DOI:** 10.3390/mi8020041

**Published:** 2017-02-01

**Authors:** Vasileios Mitrakos, Lisa Macintyre, Fiona C. Denison, Philip J.W. Hands, Marc P.Y. Desmulliez

**Affiliations:** 1 MicroSystems Engineering Centre (MISEC), School of Engineering & Physical Sciences, Heriot-Watt University, Edinburgh, EH14 4AS, UK; vm6@hw.ac.uk; 2 School of Textiles & Design, Heriot-Watt University, Galashiels, TD7 4LF, UK; l.m.macintyre@hw.ac.uk; 3 Queen’s Medical Research Institute, MRC Centre for Reproductive Health, University of Edinburgh, Edinburgh, EH16 4TJ, UK; fiona.denison@ed.ac.uk; 4 Institute for Integrated Micro & Nano Systems (IMNS), School of Engineering, University of Edinburgh, Edinburgh, EH9 3FF, UK; philip.hands@ed.ac.uk

**Keywords:** pressure sensor, capacitive sensing, polydimethylsiloxane (PDMS), tunable sensitivity

## Abstract

This article presents the design, manufacture and testing of a capacitive pressure sensor with a high, tunable performance to low compressive loads (<10 kPa) and a resolution of less than 0.5 kPa. Such a performance is required for the monitoring of treatment efficacy delivered by compression garments to treat or prevent medical conditions such as deep vein thrombosis, leg ulcers, varicose veins or hypertrophic scars. Current commercial sensors used in such medical applications have been found to be either impractical, costly or of insufficient resolution. A microstructured elastomer film of a polydimethylsiloxane (PDMS) blend with a tunable Young’s modulus was used as the force-sensing dielectric medium. The resulting 18 mm × 18 mm parallel-plate capacitive pressure sensor was characterised in the range of 0.8 to 6.5 kPa. The microstructuring of the surface morphology of the elastomer film combined with the tuning of the Young’s modulus of the PDMS blend is demonstrated to enhance the sensor performance achieving a 0.25 kPa pressure resolution and a 10 pF capacitive change under 6.5 kPa compressive load. The resulting sensor holds good potential for the targeted medical application.

## 1. Introduction

Compression garments, such as graduated compression hosiery, pressure garments and bandages, have long been known to be an effective tool to treat or even prevent medical conditions like deep vein thrombosis (DVT), leg ulcers, varicose veins or hypertrophic burn scars [1,2,3,4]. Of critical importance is the application of a moderate pressure ranging between 6 mmHg (≈ 0.8 kPa) and 50 mmHg (≈ 6.6 kPa), with different pressures and pressure gradients within this range being used to treat different conditions. To avoid subsequent medical complications and increase efficiency and efficacy of the treatment, the pressure gradient that such garments exert should ideally be monitored with a resolution of less than 0.5 kPa at the right locations on the arm, leg or body [1,5,6,7]. Unfortunately, the dynamic range, sensitivity or overall performance of current commercial sensors able to measure such low-pressure loads is limited [8,9,10,11]. Systems such as the highly sensitive capacitive sensors of Pliance X System (costing ~ $21,000) [12] are either inappropriate due to their cost, impractical to use in real-life settings, or are insufficiently sensitive or affected by the way the sensors are operated, thereby limiting the reproducibility of the results [8,11]. There is therefore an unmet clinical need for a small-sized, sensitive, practical, reliable, low-cost pressure sensor technology system capable of monitoring the low pressures required for compression garments to deliver clinically effective compression.

In recent years, a wealth of non-commercial flexible pressure sensors with the potential of integration to wearable applications have been reported, which rely on various transduction mechanisms including, but not limited to, piezoelectric [13], triboelectric [14], piezoresistive [15] and capacitive [16,17,18,19,20,21]. Capacitive pressure sensing is considered as one of the most sensitive techniques in detecting low pressures [22]. It is usually the preferred solution in low-cost applications due to the reduced complexity in both design and fabrication requirements [23] owing to the fact that the performance is solely a function of the mechanical properties and spatial dimensions of the sensor structure.

This article presents the design, manufacture and testing of a parallel plate capacitive pressure sensor. A microstructured polydimethylsiloxane (PDMS) blend film of Sylgard 184/527 with a tunable Young’s modulus of as low as 5 kPa is used as the force-sensing dielectric medium in the sensor. The combination of introducing such a PDMS blend with tunable mechanical properties and controlling the microstructured surface morphology of the thin film dielectric layer was found to enable an enhanced and fully tunable sensor performance. Preliminary results demonstrate good potential for such sensors to effectively monitor the treatment efficacy of compression garments.

## 2. Materials and Methods

**Figure 1:**
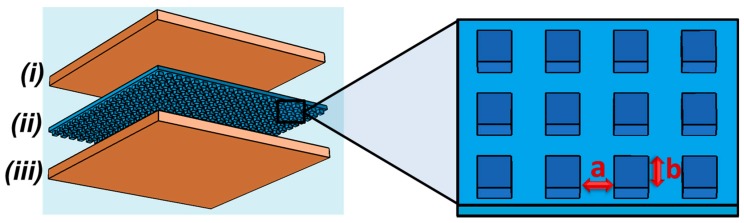
Schematic of the proposed capacitive pressure sensor where the top (i) and bottom (iii) layers are the Ti/Cu-coated glass layers used as parallel capacitor plates and to encapsulate the microstructured elastomeric blend layer (ii). The geometry of the latter layer is defined by the spacing *a* (30 and 50 μm) and dimension *b* (35 and 100 µm, respectively) of the square micro-pillar array features.

A schematic of the proposed capacitive sensor is depicted in Figure 1. The sensor has an overall size of 18 mm × 18 mm and follows a typical parallel-plate capacitor configuration. The device consists of a structured elastomeric dielectric layer encapsulated between two titanium/copper (Ti/Cu)-coated 120 µm thick glass layers that serve as capacitor plates. An array of square pillars of 35 or 100 µm size (labelled *b* in Figure 1) for a spacing of 30 and 50 μm (labelled *a*), respectively and a height of 19 µm forms the structure sitting on top of the 15 µm thick dielectric layer. Parallel plate capacitors are the most common configuration in the field of capacitive pressure sensors, and enable small, compact and cost-effective sensors with low power consumption, good direct current (DC) response and high sensitivity [24].

The mechanical deformation under compression of the intermediate dielectric medium defines the pressure sensitivity of such a pressure sensor such that:

(1) $$\Delta C=C-C_{0}=\varepsilon \cdot A\frac{\Delta d}{d\cdot \left(d-\Delta d\right)}$$

*C*_0_ is the initial capacitance value of the sensor, Δ*C* is the change of capacitance under compression, *A* is the surface area of the overlapping plates, ε is the permittivity of the dielectric medium between the two plates, *d* is the initial overall thickness of the medium and Δ*d* is the amount of compression.

For a linear elastic deformation of the dielectric medium of a Young’s modulus *E*:

(2) $$P=\frac{\Delta d}{d}E$$

and for a small deformation Δ*d* << *d*:

(3) $$\Delta C\approx C_{0}\cdot \frac{\Delta d}{d}=C_{0}\cdot \frac{P}{Ε}$$

Hence the pressure resolution Δ*C* of a capacitive pressure sensor and the resulting detectable signal output depend upon the mechanical properties of the intermediate dielectric layer through the Young’s modulus, the exerted stress and the dimensions of the sensor. The linear dependence of Δ*C* to the structure size and the requirement for small-sized capacitive devices impose a significant challenge in acquiring distinguishable capacitive signal variations to low pressure loads.

To enable large compressive deformations under low compressive loads and a high signal to noise ratio (SNR), thin film elastomers with very low Young’s modulus, such as polybutyrate (Ecoflex®, Smooth-On, Macungie, PA, USA) [16] and polyurethane [17], have been previously utilised as dielectric media (method 1). The interest in elastomer-based sensor structures stems from the potential in developing simple, cost-effective and flexible devices. However capacitive sensors based solely upon a uniform unstructured dielectric medium suffer from slow response times, due to the highly viscoelastic creep of thin-film soft elastomers [18]. Hence they require significant time to relax to their initial uncompressed state, thereby limiting their practical value.

A more judicious approach in attaining an improved pressure resolution rests in microstructuring the elastomeric dielectric medium [18,19,20,21] (method 2). This method generates effective loads that are experienced by the medium as significantly larger than the ones actually applied. In [19] this was accomplished by forming a PDMS diaphragm configuration between two Cu deposited capacitor layers on PDMS substrates; while in [20], in continuation to the work presented in [18], a PDMS micron-sized pyramidal array geometry was patterned and sandwiched between two aluminium-coated silicon wafers and, in another laminated configuration, between two indium tin oxide-coated PET substrates. The latter configuration is advantageous as the ability to control the spacing and size of the features enables a much higher and tunable sensitivity to small compressive loads (<10 kPa). The resulting device exhibits also a very fast response time of less than 1 s as the air voids allow elastomeric microfeatures to elastically deform. Moreover, the capacitor plates remain planar during compressive loading.

In this article, a combination of these two approaches (methods 1 and 2) has been explored for the manufacture of sensors with enhanced tunability in the low-pressure regime (<10 kPa). A PDMS blend with a tunable Young’s modulus of as low as 5 kPa was tested as the deformable dielectric medium. The elastomer was also structured as indicated in Figure 1. Although the microstructuring of the dielectric medium with arrays of pyramidal features with large spacing (>180 μm) and very small apex surfaces (<7 μm × 7 μm) has been demonstrated to provide a high sensitivity [20], it could however have a detrimental impact to device stability. The small resulting available surface area of such features would hinder the direct bonding of the medium to the substrate and may compromise the structural integrity of the sensor during extensive operation. The denser square micro-pillar array chosen here provides a significantly larger feature surface area to allow direct bonding of the structured layer with increased resilience to shear stresses.

An effective compressive load, *P*_eff_, can be defined as a function of the geometrical characteristics of the micro-pillar array and the tunable mechanical properties of the PDMS blend such that:

(4) $$P_{\mathrm{eff}}\propto k_{1}k_{2}P$$

(5) $$k_{1}=\frac{A}{N\cdot b^{2}}\;\mathrm{and}\;k_{2}=\frac{E}{E_{\mathrm{blend}}}$$

where *k*_1_ denotes the local compression enhancement at the surface area *b^2^* of the micro-pillar, with *N* the number of the features, and *k*_2_ the magnitude rise in compressibility of the dielectric blend medium. The effective compression load is consequently higher than the pressure exerted on an unblended dielectric medium. As a result, a higher deformation for a given load is expected with this new type of device enabling thereby a very high sensitivity of the sensor.

**Figure 2:**
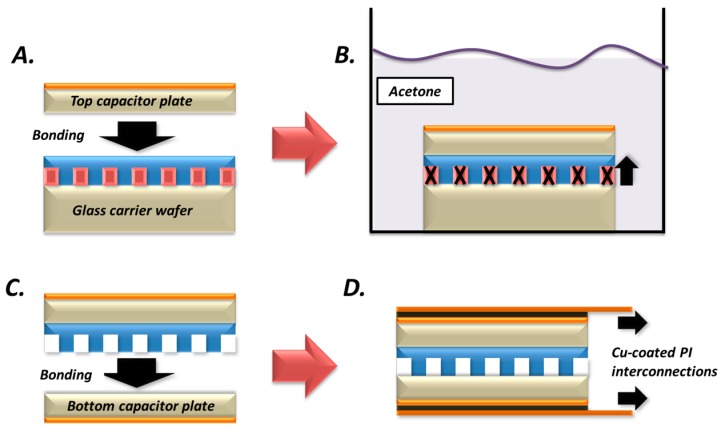
Sensor fabrication process: (**A**) Formation of a microstructured polydimethylsiloxane (PDMS) blend layer on a glass carrier wafer via a sacrificial photoresist mould and bonding of top capacitor plate Ti/Cu-coated glass layer; (**B**) detachment of carrier wafer in an acetone bath by dissolving the photoresist mould; (**C**,**D**) bonding of the bottom capacitor plate and copper-coated polyimide (Cu-PI) output interconnections.

The manufacture of the sensor follows a cost-effective process involving soft lithography for the micro-structured layer, similar to the works presented in [21,25,26,27], and a simple layer-by-layer bonding process of the individual sensor elements as depicted in Figure 2.

A 19 μm thick positive photoresist (AZ 9260, MicroChemicals GmbH, Ulm, Germany) was firstly spin-coated and developed on a glass carrier wafer, and patterned to serve as a sacrificial mould for the manufacture of the micro-pillars array. A 15 µm PDMS-based elastomer was then spin-coated into the mould and cured at 100 °C for 1 h to generate the microstructured layer of the sensor shown in panel A of Figure 2. A 18 mm ×18 mm in size and 120 μm thick borosilicate glass layer (D263®T cover glass, Schott AG, Mainz, Germany), with its top side coated with 50 nm/100 nm of Ti/Cu via e-beam evaporation, was then bonded to the surface of the elastomer to serve as one of the capacitor plates of the sensor. To enable a successful strong bond, both surfaces were treated using a hand-held corona discharge equipment (BD-20AC, Electro-Technic Products Inc., Chicago, IL, USA) for 1 min and heated at 100 °C for 1 h after contact. The corona discharge creates an oxygen plasma-rich environment, which temporarily shifts the surface of PDMS from hydrophobic to hydrophilic and enables bonding to other surfaces [28].

Demoulding of the structure, shown in panel B of Figure 2, was facilitated by an acetone bath, which dissolved the photoresist mould leaving the micro-structured layer intact and bonded onto the Ti/Cu-coated glass layer serving as the top capacitor plate of the sensor. The structure was immersed in the acetone bath for less than a minute, to avoid deterioration of the elastomer, followed by successive cleaning in Isopropyl Alcohol (IPA) and deionized (DI) water baths. The structure was then similarly bonded to a second Ti/Cu-coated glass layer to form the bottom capacitor plate of the sensor using the same surface treatment as shown in panel C of Figure 2.

A strong bond was demonstrated between the three layers using the “Scotch-tape strip test” performed on the top capacitor layer of the sensor. The micro-pillar features provided adequate surface area to enable direct bonding to the substrate and provided strong resilience to any detachment of the micro-structured layer. The strength of the bonding was also observed when shear forces were manually applied between the top and bottom capacitor plates in an effort to separate them. Detachment of the layers eventually occurred when the sensor structure was compromised through breaking the glass top and bottom glass capacitor plates under heavy loading.

A thin layer of silver epoxy was doctor-bladed on both conductive surfaces of the sensor and copper-coated thin polyimide (PI) films were bonded, as shown in panel D of Figure 2, to serve as the output interconnections of the sensor to an impedance analyser (Agilent/Hewlett Packard 4192A, Santa Clara, CA, USA). Although the Cu-coated PI films could potentially be utilised as the parallel plates of the capacitor, rigid thin Ti/Cu-coated glass layers were selected here to eliminate any unwanted influence of non-uniform buckling and bending of the sensor structure during the pressure characterisation. The glass layers also ensure that the extracted performance of the sensor is attributed solely to the impact of normal forces applied uniformly to the sensor surface.

**Figure 3:**
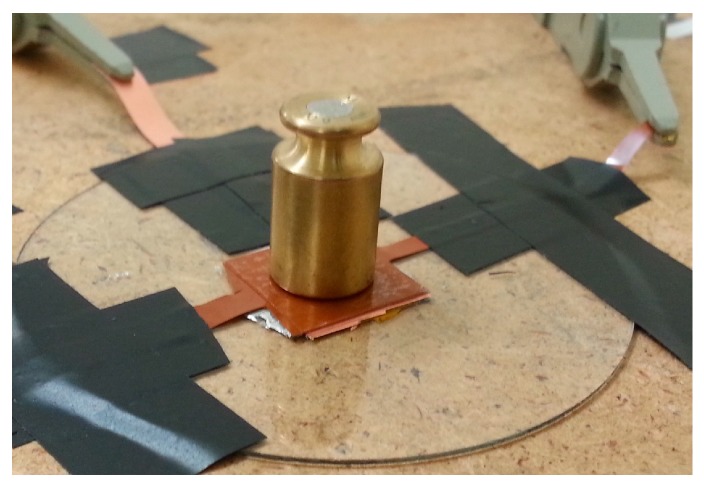
Sensor characterisation using precision weights.

Pressure measurements were manually conducted in the range of 0.8 to 6.5 kPa via a set of precision weights, ranging from 5 to 100 g as shown in Figure 3. This range corresponds to the pressure regime expected from the compression garments.

**Figure 4:**
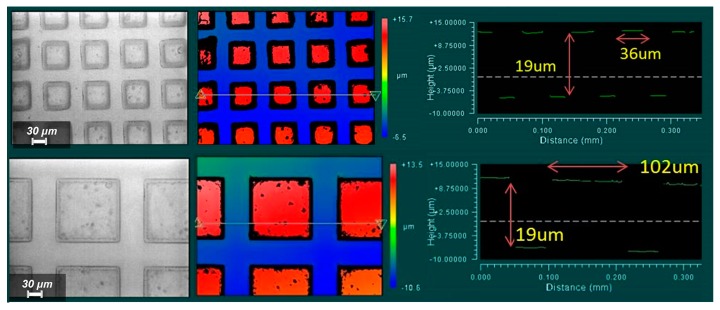
Left: Top-view photographs of the PDMS 1:5 (*E* = 130 kPa) microstructured films (top: 35 μm feature size and 30 μm spacing structure; bottom: 100 μm feature size and 50 μm spacing structure). Right: Examination of both structures under optical profilometry via a Zygo Viewmeter 5200 prior to sensor assembly.

Photographs of the dielectric layer are provided in Figure 4 alongside profilometric measurements carried out by a Zygo Viewmeter 5200 profilometer (Zygo Corporation, Middlefield, CT, USA). Good agreement was obtained between the dimensions of the desired micro-pillar array and the actual measurements (36 µm compared to 35 µm, 102 µm compared to 100 µm).

In order to explore the influence of the dielectric medium upon the sensor performance, PDMS blends of Sylgard 527 with Sylgard 184 were utilised. The adjustment of the ratio of these two constituents creates a tunable Young’s modulus with values ranging from 5 to 1.7 MPa, as reported in [29], characterised by a well-defined linear and a non- linear regime when the quantity of Sylgard 184 to Sylgard 527 either exceeds or falls below the 20% threshold respectively. Reducing the Young’s modulus of the Sylgard 184 elastomer taken alone and across that range usually involves the manipulation of the curing agent/elastomer base ratio from the recommended 1:10 value [30] to a value as low as 1:70. The latter is obtained however through partial crosslinking of the polymer resulting in unwanted diffusion of free non-crosslinked elastomer, as well as variability on the attained modulus as high as 600% [29,31,32,33,34]. However, blends based upon Sylgard 184 (*E* = 1.7 MPa) and Sylgard 527 (*E* = 5 kPa) maintain the stoichiometry of both constituents since both are prepared in their optimal stoichiometric ratio, 1:10 and 1:1 respectively, prior to being mixed together. The mixture therefore produces completely crosslinked elastomers at any given blend ratio.

In this work, three types of sensors were developed: PDMS blends of Sylgard 184 and Sylgard 527 at 1:10 and 1:5 with Young’ modulus of 50 and 130 kPa respectively; and pure PDMS Sylgard 184 with Young’s modulus 1.7 MPa [29]. Furthermore, two micro-pillar geometries, of 35 and 100 μm feature sizes and a spacing of 30 and 50 μm respectively, were also tested in combination with the above. Development of the PDMS blends was carried out in a cleanroom environment similarly to the fabrication process of the sensor described earlier, by first preparing in individual glass vials Sylgard 184 and Sylgard 527 to their optimal stoichiometric ratios, via the use of a high precision digital scale (<±0.001g), and then combing them to form the desired blend ratios. Thorough shear mixing of the constituents for each formulation was facilitated through the use of a magnetic stirrer at 500 rpm for 15 min, followed by a degassing step in a desiccator to remove trapped air pockets and produce uniform mixtures.

## 3. Results and Discussion

**Figure 5:**
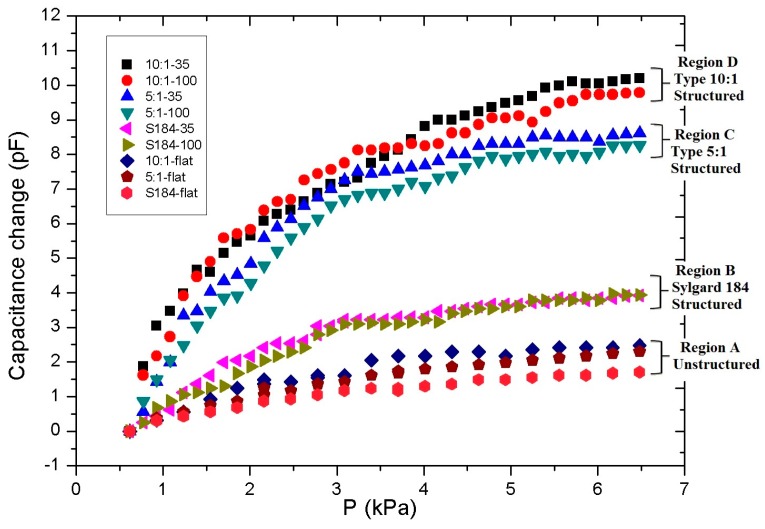
Capacitive responses of the developed pressure sensors. Region A: sensors with unstructured polymer films of pure Sylgard 184 PDMS (“S184-flat”) and Sylgard 527/184 blend types of 5:1 (“5:1-flat”) and 10:1 (“10:1-flat”). Regions B, C and D: sensors with microstructured polymeric films of pure PDMS with feature sizes of either 35 μm (“S184-35”) or 100 μm (“S184-100”), and similarly structured PDMS blend types of 5:1 (“5:1–35”, “5:1–100”) and 10:1 (“10:1–35”, “10:1–100”).

The response of the capacitive sensor was initially evaluated utilizing unstructured polymeric films as the deformable layer of the sensor (Figure 5, Region A, “S184-flat”). The change in the capacitive response recorded is small in the case of pure PDMS Sylgard 184 polymeric films (*E* = 1.7 MPa) with a 1.6 pF capacitive increase for compressive loads as high as 6.5 kPa. A similar behaviour was also observed in the case of PDMS blends of type 1:10 (*E* = 50 kPa) and type 1:5 (*E* = 130 kPa) with a maximum capacitance change of approximately 2.4 pF for both blend types over the same load (Figure 5, Region A, “10:1-flat” and “5:1-flat”). In both cases, the response of the sensor demonstrated a near-linear dependence to pressure within this dynamic range, in agreement with analysis presented earlier. A pressure resolution of 1.75 kPa was recorded, taking into account that the noise level of the experimental apparatus, and hence the lowest stable capacitance variations measured were approximately 200 fF (±100 fF).

A significantly enhanced capacitive response of the sensor was measured when a microstructured polymeric film was utilized as the deformable layer as reported also in [18,20,21]. In the case of a sensor with pure Sylgard 184 microstructured film (Figure 5, Region B “S184-35”, “S184-100”), an improved performance was recorded with a maximum capacitance value of 4 pF, for a pressure resolution of approximately 1 kPa. No substantial change in capacitance was recorded between sensors with the two pillar geometries of 35 and 100 μm dimensional sizes, possibly due to the limited capacitive resolution of the measuring system and the parasitic capacitances introduced by the output wiring.

The introduction of polymeric films of lower Young’s modulus had an even more dramatic impact on the sensor performance and sensitivity. Capacitive sensors with a microstructured film of 184/527 PDMS blend exhibited a substantially improved response, as the maximum recorded capacitance change reached 10 pF with a significantly enhanced pressure resolution down to 0.25 kPa in the case of 1:10 PDMS blend type (Figure 5, Regions C and D). A noticeable improvement in the sensor sensitivity was also observed between the type 1:5 PDMS blend (Region C) and the type 1:10 PDMS blend (Region D), as expected due to the reduced Young’s modulus of the latter, expressed as an increased response of approximately 2 pF compared to values obtained with the 1:5 PDMS blend. A slight increase in the sensor dynamic range for both blend types was also recorded amongst sensors with the two pillar geometries of 100 and 35 μm, respectively, as expected due to the reduced effective surface of the latter (Figure 5, Regions C and D, “10:1–35” vs. “10:1–100” and “5:1–35” vs. “5:1–100”). The capacitive behaviour of the sensor in this case was found to be non-linear, with a sharp response for low pressure loads below 3 kPa, followed by a capacitive increase at a slower rate, in agreement with the results reported in [18,20].

**Figure 6:**
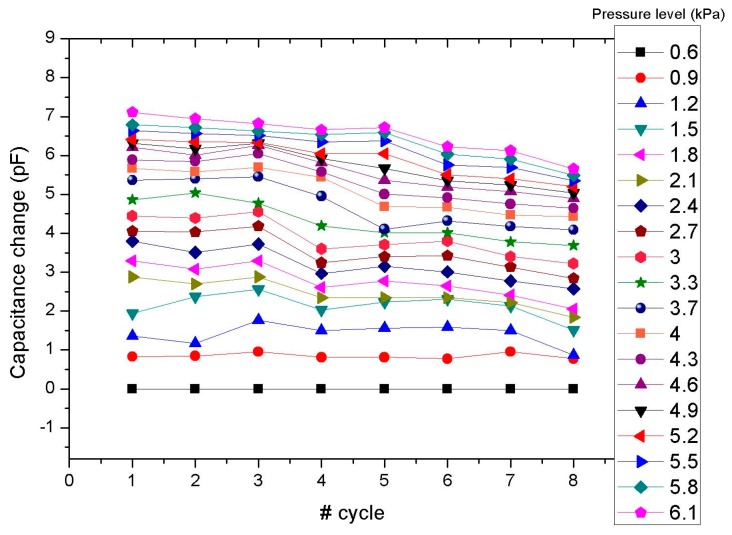
Capacitive change response of a type 5:1 pressure sensor under pressure cycling normalized over the 0.6 kPa constant load.

A good response-time to pressure cycling of less than 1 s was also observed. It was also confirmed that the microstructured PDMS blend film retracts to its original state following compressive deformation. All measurements in this case were conducted under the presence of a small load (20 grams precision weight) placed on the sensor surface, corresponding to a pressure of approximately 0.6 kPa, in order to provide a more secure site for the fast and successive addition and removal of loads. The noticeable drop in capacitance change, shown in Figure 6, is primarily attributed to the experimental apparatus. The manual positioning and stacking of precision weights involved involuntary movement and introduction of excessive loads that may have compromised the integrity of the sensor structure and disrupted the output wiring leading to additional parasitic capacitive noise reflected on the sensor response.

## 4. Conclusions

A small parallel-plate capacitive pressure sensor was developed using a simple, scalable and cost-effective fabrication process, which incorporated a microstructured 184/527 PDMS blend thin film with a tunable Young’s modulus as the force-sensing dielectric medium. A dense micro-pillar array was chosen which enables strong bonding to substrates such as Ti/Cu-coated thin glass layers utilised here, serving as the capacitor plates, leading thereby to a successful and simple sensor assembly. The introduction of the PDMS blend enabled a pressure resolution of as low as 0.25 kPa within the low-pressure range <10 kPa which, in combination with the capability to precisely control the dimensions of the features through conventional photolithography, allows a manufacturable and fully tunable sensor performance. The extracted maximum pressure resolution reported in this work was restricted by the limitations imposed by the relatively low capacitive resolution achieved by the experimental apparatus. We expect that, if a shielding technique, dedicated electronics and/or signal processing are implemented, such as in [35,36,37], much lower capacitive variations in the sub-fF regime, that correspond to pressure variations of <0.025 kPa, could be effectively recorded.

The rigid nature of the capacitor plates constitutes a drawback of the current sensor. Such a configuration does not lend itself easily to applications in wearable electronics and sensing as the device is inflexible and non-conformable. This shortcoming however can be easily overcome by eliminating the thin glass layer and utilizing flexible electrodes to serve as the capacitor plates. This could be potentially achieved by either bonding the PDMS blend microstructured layer directly to Cu-coated PI films, to allow a degree of conformability, or preferably by bonding it directly to metal-coated PDMS substrates, to allow biaxial stretching and enhanced conformability. The common issues with metal integrity (cracking) during deposition or deformation (bending/stretching) of PDMS thin films [38] would however be required to be effectively addressed, as indicated in [39,40].

Overall, the sensor tunable performance, small-size practical configuration and cost-effective fabrication process were deemed appropriate for use in compression garments or other applications requiring low of levels of pressure detection (<10 kPa) at a resolution of less than 0.5 kPa. Drawing from the above, current work is now focused in isolating the parasitic capacitive noise, developing and characterising a fully flexible sensor and integrating sensor arrays in compression garments to dynamically monitor treatment efficacy.
